# Histoplasma-Associated Mediastinal Granulomatous Disease With Superimposed Viridans Group Streptococcal Suppurative Lymphadenitis Mimicking Malignancy: A Case Report

**DOI:** 10.7759/cureus.112025

**Published:** 2026-07-03

**Authors:** Jefrina Zaman, Benedict Amalraj, Prathik Krishnan

**Affiliations:** 1 Pulmonary and Critical Care Medicine, LSU Health Shreveport, Shreveport, USA; 2 Internal Medicine, LSU Health Shreveport, Shreveport, USA

**Keywords:** antifungal therapy, bacterial mediastinitis, endobronchial ultrasound, histoplasma capsulatum, infectious mediastinal mass, lymph node abscess, mediastinal granuloma, posterior mediastinal mass, streptococcus viridans, ultrasound-guided transbronchial needle aspiration

## Abstract

A 41-year-old incarcerated male with multiple comorbidities presented with severe constitutional symptoms, including persistent fever, pleuritic chest pain, and significant unintentional weight loss. Diagnostic evaluation revealed a large, irregular posterior mediastinal mass on chest CT, characterized by calcifications, heterogeneous low-attenuation areas, and internal air locules extending to the subcarinal region adjacent to the lower thoracic esophagus. These findings initially raised concerns for an esophageal pathology complicated by potential perforation; however, an esophagram excluded esophageal rupture.

Laboratory studies demonstrated persistent thrombocytosis, hypoalbuminemia, anemia, elevated inflammatory markers, and a low-positive Histoplasma antibody titer (1:8), suggestive of fungal involvement. Further evaluation with endobronchial ultrasound-guided transbronchial needle aspiration (EBUS-TBNA) obtained purulent material from an enlarged subcarinal lymph node, supporting the presence of an active bacterial infection. Microbiological analysis demonstrated moderate growth of viridans group streptococci, which correlated with Gram-stain findings of Gram-positive cocci in chains. Although scant Staphylococcus epidermidis was also recovered, it was considered likely to represent contamination and was therefore not regarded as the primary pathogen.

Treatment with broad-spectrum intravenous antibiotics alongside systemic antifungal therapy led to significant clinical and radiological improvement. Follow-up CT imaging demonstrated marked reduction of the mediastinal mass, resolution of internal air locules, and improvement in lymphadenopathy and pleural effusions. This case report emphasizes the diagnostic complexity of infectious mediastinal masses that closely mimic malignancies. It further highlights the importance of integrating comprehensive clinical, radiological, serological, and microbiological data to establish an accurate diagnosis and guide appropriate targeted treatment.

## Introduction

Mediastinal masses pose a significant diagnostic challenge for clinicians because they encompass a diverse spectrum of underlying pathologies, ranging from inflammatory and infectious to potentially life-threatening malignancies [1]. A central difficulty in the evaluation of mediastinal masses lies in the frequent overlap between the clinical and radiological presentations of benign and malignant conditions [1]. Infectious and inflammatory processes, such as tuberculosis and histoplasmosis, can closely mimic neoplastic disorders such as lymphoma or metastatic carcinoma, presenting with constitutional symptoms, lymphadenopathy, and mass effects [2,3].

This report describes the diagnostic evaluation of a complex case involving a 41-year-old male presenting with a mediastinal mass. The diagnostic workup ultimately identified an infectious etiology, namely histoplasmosis, complicated by bacterial superinfection. Histoplasma capsulatum, an endemic fungus, can cause a broad range of thoracic manifestations, including acute pneumonia, mediastinal lymphadenopathy, mediastinal granuloma, and the more severe form, fibrosing mediastinitis [3]. Mediastinal granulomas arise from the enlargement of multiple lymph nodes, potentially creating mass-like effects that compress adjacent structures and raise concerns for malignancy [4].

## Case presentation

The patient was a 41-year-old incarcerated man with a complex medical and social history who presented with fever, chills, severe pleuritic chest pain, and marked unintentional weight loss. His medical history included Wolff-Parkinson-White (WPW) syndrome, chronic gout, gastroesophageal reflux disease (GERD), a sliding hiatal hernia, obesity treated with gastric sleeve surgery, and hypertension. He reported severe daily fevers, with a maximum temperature of 102 °F, accompanied by chest pain that was pleuritic in nature, exacerbated by deep breathing, and progressive dyspnea on exertion. These symptoms had begun approximately one month before presentation, during which he had experienced an unintentional 30-pound weight loss. There was no documented history of exposure to individuals with active tuberculosis, COVID-19, or any other contagious pulmonary infections. Although his incarceration represented a risk factor associated with congregate living, no identifiable infectious exposure was documented.

Contrast-enhanced chest CT revealed a large, irregular posterior mediastinal mass with calcifications, heterogeneous low-attenuation areas, and intralesional air, extending to the subcarinal region and appearing inseparable from the lower thoracic esophagus. Right hilar (2 × 1.6 cm) and right lower paratracheal (1.7 × 1.4 cm) nodes were enlarged, with small bilateral pleural effusions and mild basal atelectasis. The combination of intralesional gas and esophageal contiguity prompted concerns for an esophageal malignancy with a possible contained perforation.

Clinical findings

Physical examination findings provided further clues, including vital signs such as persistent fever and tachycardia. Respiratory examination demonstrated normal breath sounds without wheezing or rhonchi, and there was no respiratory distress. Cardiovascular examination revealed normal heart sounds without murmurs, gallops, or rubs. On abdominal examination, mild tenderness was noted in the left upper quadrant (LUQ) and left lower quadrant (LLQ).

Laboratory data

Laboratory investigations on admission revealed a markedly elevated white blood cell count of 14.24 ×10⁹/L, consistent with leukocytosis. Platelet count was significantly increased at 711 ×10⁹/L, indicating pronounced thrombocytosis. Hemoglobin was measured at 13.0 g/dL, and hematocrit was 38.6%, both at the lower end of the normal range. The patient demonstrated hypoalbuminemia, with an albumin level of 3.1 g/dL. Inflammatory markers were notably elevated, with a C-reactive protein (CRP) of 6.10 mg/L and an erythrocyte sedimentation rate (ESR) of 55 mm/hr. Procalcitonin was 0.10 ng/mL, indicating a low risk for systemic bacterial infection. Other laboratory parameters, including renal function and liver enzymes, were within or near normal limits. Importantly, tests for other primary differential diagnoses, such as HIV and tuberculosis, yielded negative results. A comprehensive summary of the admission laboratory findings is provided in Table 1.

**Table 1:** Summary of the admission laboratory findings This table summarizes the key laboratory findings for the patient on the day of hospital admission. The results are organized into three main categories: complete blood count (CBC), comprehensive metabolic panel (CMP), and inflammatory markers (ESR, CRP, procalcitonin) WBC: white blood cells; RBC: red blood cells; ANC: absolute neutrophil count; BUN: blood urea nitrogen; ALP: alkaline phosphatase; AST: aspartate aminotransferase; ALT: alanine aminotransferase; eGFR: estimated glomerular filtration rate; CRP: C-reactive protein; ESR: erythrocyte sedimentation rate

Component	Result	Reference range
WBC (x10³/µL)	14.24	4.0 – 11.0
RBC (x10⁶/µL)	4.26	4.5 – 5.9
Hemoglobin (g/dL)	13.0	13.5 – 17.5
Platelets (x10³/µL)	711	150 – 400
ANC (x10³/µL)	11.1	1.8 – 7.7
Immature grans (Abs)	0.11	0.00 – 0.04
Lymphocytes (x10³/µL)	2.3	1.0 – 4.8
Monocytes (x10³/µL)	0.7	0.3 – 1.0
Eosinophils (x10³/µL)	0.0	0.0 – 0.5
Basophils (x10³/µL)	0.08	0.00 – 0.20
Granulocytes (%)	77.6	38 – 73
Lymphocytes (%)	15.8	18 – 48
Monocytes (%)	5.1	4 – 15
Eosinophils (%)	0.1	0 – 8
Basophils (%)	0.6	≤1.9
Sodium (mmol/L)	140	136 – 145
Potassium (mmol/L)	4.3	3.5 – 5.1
Chloride (mmol/L)	107	98 – 110
CO_2_ (mmol/L)	26	22 – 29
Glucose (mg/dL)	140	70 – 110
BUN (mg/dL)	8	6 – 20
Creatinine (mg/dL)	0.9	0.5 – 1.4
Calcium (mg/dL)	9.4	8.7 – 10.5
Total protein (g/dL)	7.2	6.0 – 8.3
Albumin (g/dL)	3.1	3.5 – 5.0
Total bilirubin (mg/dL)	0.6	0.1 – 1.0
ALP (U/L)	92	40 – 129
AST (U/L)	25	10 – 40
ALT (U/L)	41	10 – 44
eGFR (mL/min/1.73m²)	>60	> 60
Anion gap (mmol/L)	7	8 – 16
Procalcitonin (ng/mL)	0.10	< 0.25
CRP (mg/L)	6.10	< 3.0
ESR (mm/hr)	55	0 – 20

Microbiology

Microbiologic evaluation targeted infectious causes of the mediastinal mass and systemic symptoms. Blood samples were submitted for rapid PCR-based identification, and tissue and aspirated fluid from the involved lymph node were obtained via endobronchial ultrasound-guided transbronchial needle aspiration (EBUS-TBNA). Both tissue and fluid cultures grew viridans streptococci (in moderate quantities) and Staphylococcus epidermidis (in scant quantities). The tissue Gram stain showed numerous leukocytes, but no organisms were visualized, whereas the fluid Gram stain demonstrated Gram-positive cocci in chains, morphologically consistent with streptococci. The simultaneous detection of Staphylococcus epidermidis was attributed to contamination.

The broader workup for alternative pathogens was unrevealing: fungal cultures were negative, no acid-fast bacilli were isolated, and serologies for Francisella tularensis and Brucella spp. were negative. Serum Histoplasma antibodies were positive, with a titer of 1:8. Given the patient’s significant occupational exposure risk for Histoplasma capsulatum, empiric therapy was initiated to provide coverage for both fungal and possible bacterial etiologies.

Imaging findings

Chest imaging studies, including CT (Figures 1, 2) and an esophagram, were essential in diagnosing and characterizing the patient's complex mediastinal pathology. The initial CT scan of the chest with contrast on admission revealed a large, irregular mass situated in the posterior mediastinum. Several concerning features were noted: the mass contained calcifications with internal low-attenuation regions, as well as locules of air within the mass (Figure 1). It extended inferiorly to the subcarinal region and appeared inseparable from the lower thoracic esophagus near the gastroesophageal junction. Associated findings included an enlarged right hilar lymph node (2.0 × 1.6 cm; yellow asterisk in Figure 1) and a borderline enlarged right lower paratracheal lymph node measuring 1.7 × 1.4 cm. Additionally, small bilateral pleural effusions (greater on the right) with mild bilateral basal atelectasis. Given the mass's appearance and location adjacent to the esophagus, with internal air, there was initially concern for a neoplastic process, such as esophageal cancer, with a possible contained perforation. A complete fluoroscopic esophagram revealed a normal swallowing mechanism and normal esophageal caliber, without any strictures. Crucially, there was no extravasation of contrast medium, ruling out an esophageal perforation.

**Figure 1:**
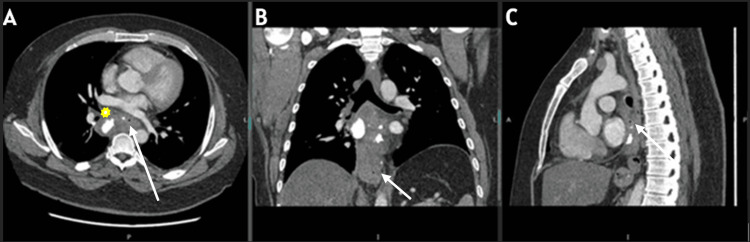
Contrast-enhanced CT images - 1 (A) Axial, (B) coronal, and (C) sagittal contrast-enhanced CT images demonstrate an enlarged right hilar lymph node (yellow asterisk; approximately 2.0 × 1.6 cm) adjacent to the right main bronchus and pulmonary artery. A borderline enlarged right lower paratracheal lymph node measuring approximately 1.7 × 1.4 cm is also present. These nodal findings accompany the posterior mediastinal mass and, in the context of granulomatous infection, favor reactive or suppurative lymphadenitis over metastatic disease CT: computed tomography

**Figure 2:**
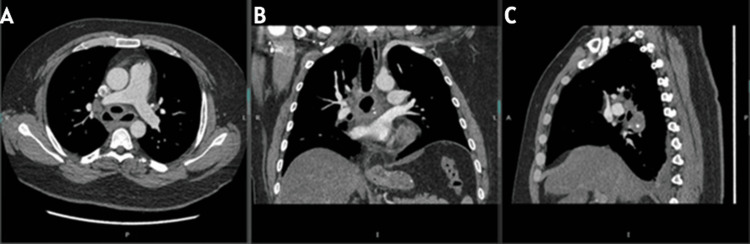
Contrast-enhanced CT images - 2 (A) Axial, (B) coronal, and (C) sagittal contrast‑enhanced CT images depict an enlarged right hilar lymph node measuring approximately 20 × 16 mm, situated adjacent to the right main bronchus and pulmonary artery. A borderline enlarged right lower paratracheal node is also appreciable on the coronal view. These nodal enlargements accompany the posterior mediastinal mass and, in the context of granulomatous infection, favour reactive or suppurative lymphadenitis over metastatic disease CT: computed tomography

Bronchoscopy findings

A mediastinal mass was identified on imaging and investigated for concern of malignancy. The patient underwent bronchoscopy with convex EBUS-TBNA. During the procedure, direct visualization of the airways showed patent airways without evidence of external compression with normal-appearing mucosa. Upon surveillance of the subcarinal region with convex EBUS, an enlarged and irregularly appearing lymph node was identified at station 7, which corresponded to the abnormality seen on CT. Seven transbronchial needle aspirations were performed under real-time ultrasound guidance targeting the station 7 lymph node. Figure 3 illustrates the initial evaluation and ultrasound findings during EBUS. The most significant finding during the aspiration was the return of purulent-appearing material from the node. The aspirated purulent material was sent for comprehensive analysis, including microbiological and flow cytometry studies. 

**Figure 3:**
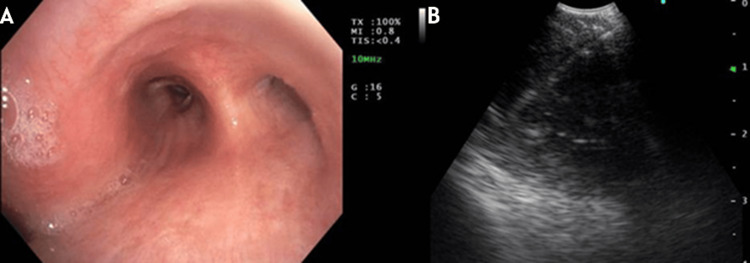
Bronchoscopy results (A) Bronchoscopic view demonstrating a patent airway with normal-appearing mucosa and no external compression, confirming intraluminal integrity despite the large posterior mediastinal mass. (B) Real‑time convex endobronchial ultrasound image revealing an enlarged, heterogeneous, hypoechoic lymph node consistent with suppurative lymphadenitis. Subsequent transbronchial needle aspiration of this node returned purulent material that cultured viridans group streptococci

Results

The initial presentation of our patient was alarming, characterized by severe constitutional symptoms, including high fevers, pleuritic chest pain, and significant weight loss, alongside laboratory evidence of profound systemic inflammation (initial leukocytosis, elevated CRP/ESR, and marked thrombocytosis) and chronic illness (anemia and hypoalbuminemia). The admission chest CT scan intensified concern, revealing a large, irregular posterior mediastinal mass with calcifications, internal air locules, and low-attenuation areas, associated with enlarged lymph nodes and pleural effusions, findings highly suspicious for malignancy and potentially complicated by esophageal perforation. However, esophageal perforation was ruled out. While broad infectious workups, including HIV, tuberculosis, tularemia, and brucellosis testing, were negative, a low-positive Histoplasma yeast antibody titer (1:8) provided the first clue suggesting a possible underlying fungal etiology.

The subsequent EBUS-TBNA procedure targeting the enlarged subcarinal (station 7) lymph node proved pivotal in clarifying the diagnosis. The TBNA yielded grossly purulent material, a finding strongly indicative of an active infection. This was corroborated by microbiology results from the aspirate, which demonstrated moderate growth of viridans group streptococci (consistent with Gram stain findings) and scant Staphylococcus epidermidis, confirming bacterial lymphadenitis with abscess formation.

The diagnostic picture was confirmed by follow-up CT imaging obtained approximately two weeks after the initiation of treatment. This scan demonstrated marked interval improvement, with a decrease in the size of the mediastinal mass and associated lymph nodes, complete resolution of the internal air locules, and clearance of pleural effusions and atelectasis. The favorable clinical response to antimicrobial therapy strongly supported an infectious or inflammatory etiology rather than malignancy. The final favored diagnosis was Histoplasma-associated granulomatous mediastinal disease complicated by superimposed viridans group streptococcal suppurative lymphadenitis and abscess formation. This underlying Histoplasma-related inflammation was accompanied by superimposed bacterial lymphadenitis or abscess formation, with malignancy being less likely.

A follow-up CT scan of the chest, abdomen, and pelvis revealed a marked improvement in the mediastinal process: the large mass appeared smaller, and more critically, the previously observed internal air locules had resolved. Furthermore, the enlarged right hilar and paratracheal lymph nodes were of reduced size. The imaging initially revealed findings highly suspicious for malignancy, complicated by perforation, but the condition evolved significantly over two weeks with appropriate antibiotic and antifungal treatment. 

The patient was treated with cefepime 2 g IV every eight hours, metronidazole 500 mg IV every eight hours, and itraconazole 200 mg twice daily, with itraconazole planned for a total six-month course. Follow-up CT imaging approximately two weeks after treatment initiation demonstrated an interval decrease in the posterior mediastinal mass and associated lymphadenopathy, resolution of internal air locules, and improvement in pleural effusions and atelectatic changes. Specific follow-up documentation regarding weight change and chest pain severity was not available. Follow-up CT images were not available for inclusion as figures, which is a limitation of this report.

## Discussion

This case report of a 41-year-old male with a complex medical history underscores the significant diagnostic challenge posed by mediastinal masses that can closely resemble malignancy. The patient's initial presentation included severe constitutional symptoms, namely high-grade fevers, debilitating pleuritic chest pain, and substantial weight loss. This was accompanied by initial CT findings of a large, irregular posterior mediastinal mass with calcifications, low-attenuation areas, and air locules, which raised strong suspicion for a primary malignancy or a complicated infectious process, such as an abscess or esophageal perforation with resulting mediastinitis. Common causes of bacterial mediastinitis include esophageal rupture, direct extension from deep neck or thoracic infections, and post-surgical complications. The presence of air locules initially heightened concern for perforation, which was ultimately excluded by an esophagram [5]. Furthermore, such infections may progress to sclerosing mediastinitis [6]. The differential diagnosis remained broad, encompassing lymphoma, metastatic disease, granulomatous infections (such as histoplasmosis and tuberculosis), and primary bacterial abscesses or lymphadenitis [7,8].

The diagnostic pathway pivoted significantly based on integrated findings. While laboratory results confirmed severe systemic inflammation (elevated CRP/ESR, thrombocytosis) and chronic illness (anemia, hypoalbuminemia), the positive Histoplasma yeast antibody, albeit at a low titer of 1:8, provided the first specific clue towards an infectious etiology, supported by the CT finding of calcifications often seen in healed granulomatous disease. The EBUS-TBNA procedure was critical, as it yielded samples negative for malignant cells on preliminary cytology and produced purulent material from the subcarinal lymph node. This finding was significant, providing direct evidence of an active, suppurative infection. Subsequent microbiology confirmed this, isolating moderate viridans group streptococci (consistent with the Gram stain) and a few Staphylococcus epidermidis, which strongly suggested bacterial lymphadenitis or abscess formation, likely driven by the Streptococcus, possibly superimposed on an underlying Histoplasma-related granulomatous process.

The patient's clinical course and response to treatment further solidified the infectious diagnosis. Despite initial management (including likely antibiotics post-EBUS given the purulent finding), the patient required readmission due to persistent fevers. This prompted the initiation of broad-spectrum intravenous antibiotics (cefepime 2 gm IV every eight hours and metronidazole 500 mg IV every eight hours) concurrently with systemic antifungal therapy targeting the suspected histoplasmosis (Itraconazole 200 mg BID, started and planned for a six-month total duration). The follow-up CT scan, obtained shortly after the intensified regimen was initiated, demonstrated marked radiological improvement, including a reduction in the size of the mediastinal mass and lymph nodes, as well as resolution of air locules and pleural effusions. These findings provided compelling evidence that combined antibacterial and antifungal therapy was effectively treating the underlying pathology.

The patient's comorbidities, including a history of incarceration (a potential risk factor for Histoplasma exposure) and chronic conditions, led to this severe, complex presentation involving both fungal and bacterial elements. Histoplasmosis and other infections have been associated with mediastinal masses [9,10]. Rare bacterial superinfection of Histoplasma-associated mediastinal granulomatous disease has been described in the literature [11]. This report highlights the importance of a comprehensive, multidisciplinary approach, which integrates clinical progression, serial imaging, specific serological markers, and invasive sampling with microbiological analysis, particularly when initial presentations are misleading [8].

## Conclusions

This report highlights the diagnostic complexity of a posterior mediastinal mass in a 41-year-old incarcerated male with multiple comorbidities who presented with severe constitutional symptoms, pleuritic chest pain, and significant unintentional weight loss. Initial imaging findings were highly concerning for malignancy or a complicated esophageal process; however, subsequent evaluation supported an infectious and inflammatory etiology. Key findings included a low-positive Histoplasma yeast antibody titer of 1:8, mediastinal calcifications suggestive of prior granulomatous disease, benign cytology, and EBUS-TBNA of the subcarinal lymph node yielding grossly purulent material with growth of viridans group streptococci. Together, these findings favored suppurative bacterial lymphadenitis or abscess formation, likely superimposed on Histoplasma-associated granulomatous mediastinal disease. The patient’s clinical course and interval radiologic improvement following broad-spectrum intravenous antibiotics and prolonged itraconazole therapy further supported an infectious or inflammatory process rather than malignancy. This report demonstrates that endemic mycoses, particularly when complicated by bacterial superinfection, may closely mimic malignant mediastinal disease. Accurate diagnosis requires integration of clinical presentation, laboratory findings, serology, imaging characteristics, invasive sampling, microbiology, cytology, and treatment response. Such a multidisciplinary approach is essential for distinguishing infection from malignancy and guiding appropriate management in complex presentations of mediastinal disease.

## References

[1] Naeem F, Metzger ML, Arnold SR, Adderson EE (2015). Distinguishing benign mediastinal masses from malignancy in a histoplasmosis-endemic region. J Pediatr.

[2] Almeida PT, Heller D (2025). Anterior Mediastinal Mass. Anterior Mediastinal Mass.

[3] Chang TF, Ang JY (2020). A case of a mediastinal mass in a teenager causing chest pain, difficulty breathing, and emesis: a rare complication of a relatively common disease. Clin Pediatr (Phila).

[4] Barros N, Wheat JL, Hage C (2023). Pulmonary histoplasmosis: a clinical update. J Fungi (Basel).

[5] Kappus S, King O (2025). Mediastinitis. King, Mediastinitis, in StatPearls.

[6] Marino M, Ascani S (2019). An overview on the differential diagnostics of tumors of the anterior-superior mediastinum: the pathologist's perspective. Mediastinum.

[7] Ghigna MR, Thomas de Montpreville V (2021). Mediastinal tumours and pseudo-tumours: a comprehensive review with emphasis on multidisciplinary approach. Eur Respir Rev.

[8] Ahuja J, Strange CD, Agrawal R, Erasmus LT, Truong MT (2023). Approach to imaging of mediastinal masses. Diagnostics (Basel).

[9] Zeanah CH, Zusman J (1979). Mediastinal and cervical histoplasmosis simulating malignancy. Am J Dis Child.

[10] Schweigert M, Dubecz A, Beron M, Ofner D, Stein HJ (2013). Pulmonary infections imitating lung cancer: clinical presentation and therapeutical approach. Ir J Med Sci.

[11] Emoto JL, Wittler RR (2024). A case report of a mediastinal granuloma related to histoplasmosis with Streptococcus dysgalactiae subspecies equisimilis superinfection resulting in abscess and subsequent pericarditis in a pediatric patient. Kans J Med.

